# A UK Biobank Study on Genetic Variants in Pattern-Recognition Receptor (PRR) Signaling Indicates Self-Perpetuatin Inflammation of Cholesteatoma

**DOI:** 10.3390/jpm16020094

**Published:** 2026-02-05

**Authors:** Mohannad Almomani, Ioannis Vlastos, Kalliopi Gkouskou, Nikolaos Drimalas, Jiannis Hajiioannou

**Affiliations:** 1 Evangelismos General Hospital, Ipsilantou 45-47, 10676 Athens, Greece; 2 Department of Otolaryngology-Head and Neck Surgery, Medical School, University of Thessaly, 3 Panepistimiou Str., 41500 Larissa, Greece; ioanchat@uth.gr

**Keywords:** cholesteatoma, pattern-recognition receptors, genetic polymorphisms, inflammation, UK biobank, genetic risk score

## Abstract

**Background**: Acquired cholesteatoma is a chronic inflammatory middle ear disease characterized by keratinizing squamous epithelium overgrowth and bone erosion. While the upregulation of pattern-recognition receptor (PRR) signaling has been consistently observed, it remains unclear whether this reflects a secondary response to microbial infection or a primary dysfunction driven by genetic predisposition. **Methods**: Using the UK Biobank, we analyzed 678 individuals with cholesteatoma (ICD-10: H71) among 502,164 participants. Candidate genes implicated in cholesteatoma-related inflammatory pathways (n = 17) were selected, and 147 polymorphisms were studied. Gene-specific genetic risk scores (GRSs) were calculated for cholesteatoma patients (GRSchol) and the general UK Biobank population (GRSpop). The difference (ΔGRSchol-GRSpop) was used to assess the relative contribution of each gene. **Results**: Genes with the highest ΔGRS were *IL6*, *TREM1*, *IL1R1*, *IL1A*, *HIF1A*, *ID1*, *RAGE*, and *TNFA*. These genes represent key downstream mediators and amplifiers of PRR signaling rather than the receptors themselves. Variants in cytokine genes (*IL6*, *IL1R1*, *IL1A*, and *TNFA*) may enhance inflammatory signaling and bone resorption; *Trem1* amplifies TLR responses; *RAGE* sustains sterile DAMP-driven inflammation, while *HIF1A* and *ID1* implicate hypoxia, tissue remodeling, and keratinocyte proliferation in disease persistence. **Conclusions**: Our findings suggest that cholesteatoma pathogenesis may not be driven solely by microbial activation of PRRs but rather by genetic variants that amplify and sustain downstream inflammatory responses. This supports a model of cholesteatoma as a disease of self-perpetuating inflammation triggered by diverse stressors, including microbial and non-microbial insults. These insights may inform preventive strategies targeting environmental stressors, as well as therapeutic approaches using biologics to interrupt chronic inflammatory amplification in cholesteatoma.

## 1. Introduction

Cholesteatoma is an expansile, destructive epithelial lesion of the temporal bone, affecting approximately 3–15 per 100,000 adults and children [1,2]. Formed by keratinizing squamous epithelium in the middle ear and/or mastoid, it progressively accumulates keratin debris, often accompanied by inflammation. It is an important global cause of potentially preventable hearing loss. Patients experience substantial reductions in quality of life and face risks of serious complications, including facial paresis, profound hearing loss, and intracranial abscess [3,4]. An upregulated pattern-recognition receptor (PRR) signaling has been reported in numerous studies [5,6,7,8,9], which is expected as the disease is associated with tissue destruction, cell death, and microbial infection [10].

PRRs are proteins expressed predominantly by cells of the innate immune system, such as dendritic cells, macrophages, and neutrophils, as well as by epithelial cells [11]. They recognize two major classes of molecules: pathogen-associated molecular patterns (PAMPs), which originate from bacteria, and damage-associated molecular patterns (DAMPs), derived from stressed or dying cells as well as from degraded extracellular matrix components [10].

Several studies have demonstrated the upregulation of PRR-related pathways in cholesteatoma [5,6,7,8,9,12,13]. Particularly, the PAMP Lipopolysaccharide (LPS), which originates from Gram (−) bacteria, has been consistently linked to MEC formation and growth in vitro or in vivo in patients [8,9] and in an animal model [7]. However, it remains unclear whether this upregulation reflects a secondary response—for instance, triggered by microbial infection—or whether it points to a primary dysfunction, such as genetic variants predisposed to excessive Th1-driven inflammation, causing changes in host–microbe interactions, which can trigger dysbiosis and destructive inflammation [14]. In the latter case, a self-perpetuating inflammatory environment may be established, fueling chronic disease progression.

The increased expression of inflammatory mediators alone does not clarify whether this represents a cause or consequence of disease. Investigation of genetic variants in key PRR signaling genes may therefore shed light on potential predisposing factors for cholesteatoma. An increased frequency of certain variants in patients may reveal pathways implicated in the onset and persistence of disease.

The present study aims to systematically examine genetic variants in genes encoding major components of PRR signaling, to compare their frequencies with those observed in the general population, and to calculate gene-specific genetic risk scores. Ultimately, our goal is to identify genetic determinants that may contribute to cholesteatoma pathogenesis by sustaining a chronic inflammatory milieu.

## 2. Materials and Methods

**Figure 1:**
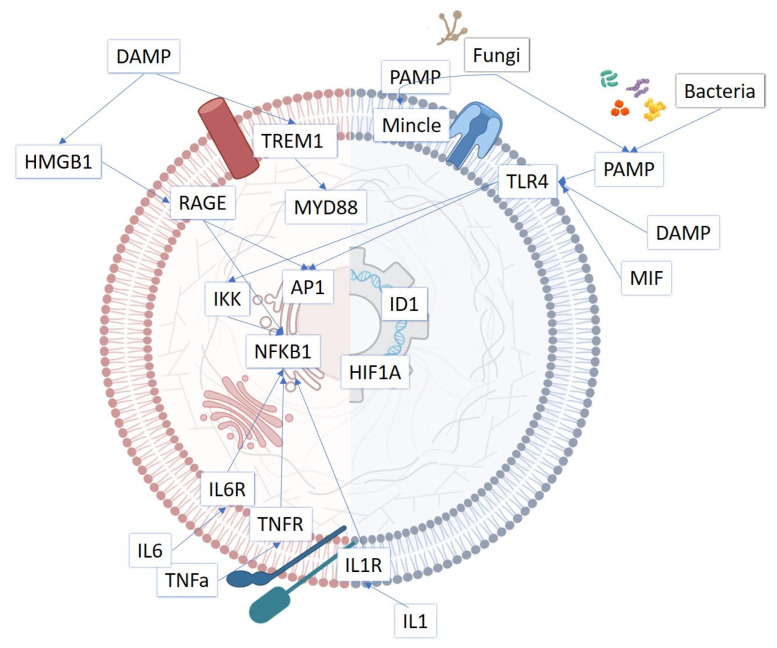
The proinflammatory intra and extracellular signaling that has already been studied in cholesteatoma disease, either in vivo or in vitro. Bacteria, fungi, keratin debris, LPS, and other PAMPs activate PPRs, such as *TLR4*, for cytokine production. In our study, variants were more frequently observed in *IL6*, *TREM1*, *IL1R1*, *IL1A*, *HIF1A*, *ID1*, *RAGE*, and *TNFA* genes in cholesteatoma patients compared with the general UK Biobank population. The majority of these genes, with the exception of *TREM* and *RAGE*, are not directly related to DAMPs and PAMPs but act in the nucleus as regulatory factors of inflammation.

For the purposes of the study, data from the UK Biobank (UKBB) were utilized. The UKBB is a large-scale biomedical database and research resource containing in-depth genetic, health, and lifestyle information from approximately 500,000 participants across the United Kingdom, aged between 40 and 69 years at the time of recruitment (2006–2010). We accessed genotype and phenotypic data from the UK Biobank under approved application number 117308, focusing on genetic variants within candidate genes implicated in key inflammatory pathways in cholesteatoma (*MYD88*, *RAGE*, *NFKB1*, *IL1R1*, *IL1B*, *IKK Ab*, *ID1*, *AP1*, *HIF1A*, *IL6*, *TLR4*, *HMBG*, *TNFA*, *MIF*, *MINCLE*, *TREM*, and *ILA*). A schematic diagram with these factors can be found in Figure 1 (adapted from a previous review) [10]. Candidate genes were selected based on converging evidence from in vivo, ex vivo, and in vitro studies demonstrating their involvement in cholesteatoma-related inflammation, epithelial proliferation, or osteolysis. [10]. This ensured that the analysis focused on biologically plausible pathways directly implicated in disease mechanisms rather than exploratory genome-wide associations.

All data were accessed through the DNAnexus platform. The use of the study subject information in the database was anonymous. A clinical filter for H71 (cholesteatoma) based on ICD-10 record diagnosis codes was applied. In total, 678 patients with cholesteatoma were identified among 502,164 participants.

**Scheme 1:**
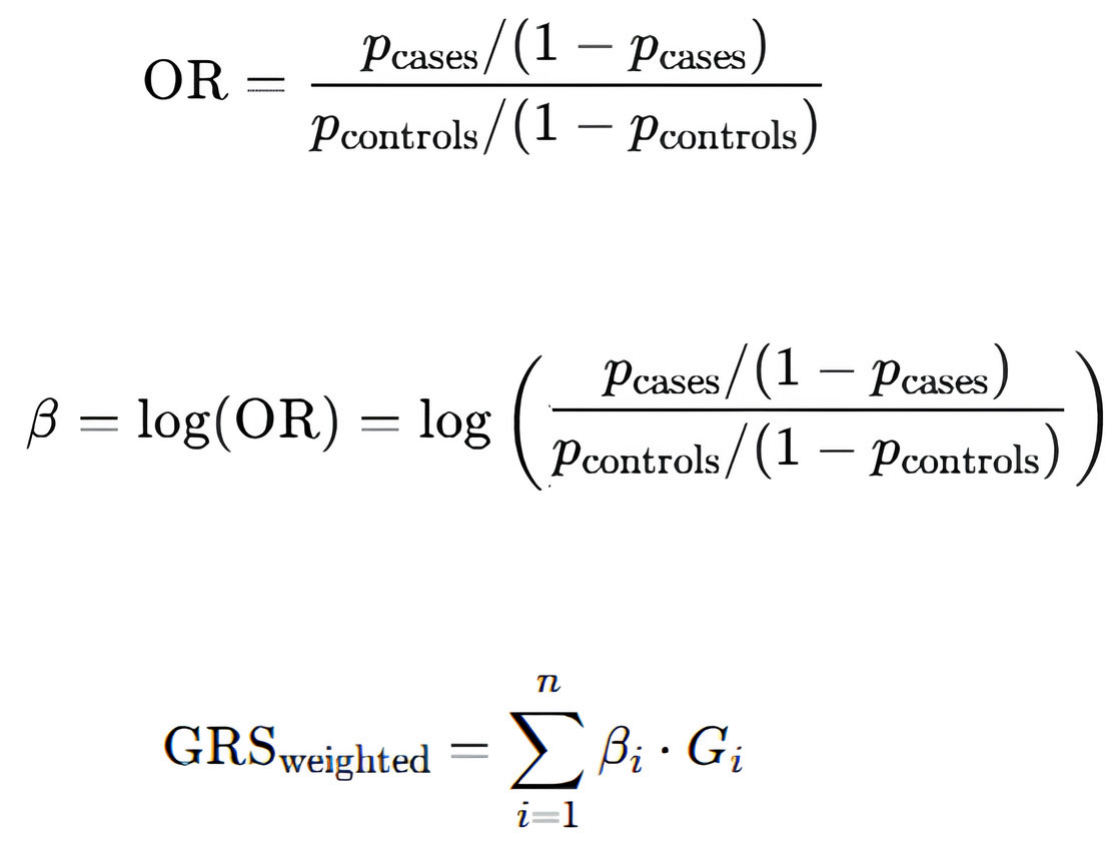
β-coefficient, which indicates the effect size of each risk allele, can be calculated by the allele frequencies of the cohort (*pcases*) and the population (*pcontrols*). *Gi* is the genotype (0,1,2) or, in other words, the number of risk alleles. Thus, *βi∙Gi* estimates the average genetic contribution of SNP i to the disease risk, and the GRS is the sum of the genetic contributions of risk alleles.

For the formulation of a gene-specific genetic risk score (GRS), genotypes were extracted and downloaded as a .csv file for each gene of interest, containing allele frequencies in the cohort and the general population. The weighted GRS for each gene was calculated as the sum of the number of risk alleles multiplied by their respective β-coefficients, where β represents the effect size (the natural logarithm of the odds ratio) for each single-nucleotide variant (SNV). β-coefficients have been calculated using the allele frequencies of the UK Biobank. Scheme 1 shows the equations used for this calculation. In more detail, to derive these β-coefficients, odds ratios (ORs) were first estimated using allele frequencies in the cholesteatoma cohort (p_cases) and the general population (p_controls). Because no prior studies have defined pathogenic variants or assigned differential weights to heterozygous versus homozygous states in these genes, we applied a standard polygenic-risk-score approach and used the log(OR) of each SNV as its β-coefficient. The additive properties of β-coefficients on the log-odds scale make them suitable for constructing risk scores, whereas ORs themselves cannot be directly combined. SNVs with an allele frequency below 1%, as well as those in linkage disequilibrium (i.e., alleles of genetic loci in close proximity with strongly correlated frequencies, e.g., LD; r^2^ > 0.8), were excluded from the GRS calculation [15].

Genetic risk scores for cholesteatoma patients (GRSchol) as well as the total population (GRSpop) of the UK biobank were calculated for each gene. The differences between them (ΔGRSchol-GRSpop) were then compared. An increased gene-specific GRS indicates that variants of the gene of interest are relatively more common in cholesteatoma patients compared to the rest of the genes. To obtain statistically valid inference, we applied a gene-level nonparametric bootstrap. The resulting bootstrap distribution was used to derive the mean GRS and the 95% confidence interval. This may suggest a greater contribution of that gene to the pathogenesis of cholesteatoma.

In addition, we conducted covariate-adjusted regression analyses utilizing sex, age, deprivation, and tobacco use as independent variables. Variants with increased β-coefficients of the genes that exhibited the largest differences in genetic risk scores were utilized for the regression analyses.

## 3. Results

A total of 147 genetic variants in 17 key genes involved in PRR signaling pathways of inflammation have been studied. Supplementary Table S1 shows these variants and their β-coefficient. Table 1 shows the genes of interest and their ΔGRSchol-GRSpop scores. Positive ΔGRS values indicate that risk alleles were relatively enriched in cholesteatoma patients compared with the general UK Biobank population, suggesting a potential contribution of these genes to disease susceptibility or persistence. Genes with the greatest score were *IL6*, *TREM*, *IL1R1*, *ILA*, *HIF1A*, *ID1*, *RAGE*, and *TNFA*. Bootstrap-derived confidence intervals confirmed that *IL6*, *TREM1*, *IL1R1*, *IL1A*, *HIF1A*, *ID1*, *RAGE*, and *TNFA* consistently ranked highest, with non-overlapping CIs compared to lower-ranking genes. Notably, the genes showing the largest ΔGRS were those encoding master regulators of inflammation *(IL6*, *IL1A*, and *TNFA*), amplifiers of PRR signaling (*TREM1*, *RAGE*), and regulators of epithelial proliferation and hypoxia response (*ID1*, *HIF1A*). The convergence between statistical enrichment and biological plausibility strengthens the interpretation that these pathways may collectively sustain a self-perpetuating inflammatory microenvironment.

Examples of the regression analyses in selected genetic variables are shown in Table 2, and more specifically, the association of rs139240442 (*IL6*) and rs140764737 (*TREM10*) with demographic, lifestyle, and socioeconomic variables. None of the examined factors showed a statistically significant association with these genetic variants.

## 4. Discussion

Several PRR signaling pathways have been reported to play a role in cholesteatoma pathogenesis. Toll-like receptors (TLRs) are the most extensively studied, particularly TLR2 and *TLR4*, which are upregulated in cholesteatoma epithelium and perimatrix [16,17,18]. Their activation by bacterial PAMPs (such as lipopolysaccharide or peptidoglycan) leads to MyD88-dependent NF-κB activation and production of cytokines, including TNF-α, IL-1β, and IL-6, which drive chronic inflammation and bone resorption [6,19].

Evidence also supports a role for NOD-like receptor (NLR) signaling, especially the NLRP3 inflammasome, which promotes the caspase-1-mediated maturation of IL-1β and IL-18 [20,21]. This pathway is thought to contribute to sustained inflammation and osteoclast activation in cholesteatoma. In addition, *RAGE* (a receptor sensing DAMPs) and high-mobility group box 1 protein (HMGB1) signaling have been described, linking epithelial stress and extracellular matrix breakdown to a self-perpetuating inflammatory loop [12]. Overall, these findings suggest that both microbial triggers (via TLRs) and endogenous danger signals (via NLRs and *RAGE*) converge to create a chronic, self-sustaining inflammatory microenvironment in cholesteatoma.

In particular, microbial factors seem to exert a significant role in cholesteatoma formation, progression, and prognosis. Recent studies using metagenomics approaches detected bacterial DNA in all cholesteatoma patients [22], whereas the application of specific drugs according to antibiotic susceptibility testing improved the rate of dry ear after surgery significantly [23,24]. Nevertheless, the molecular science of cholesteatoma pathogenesis seems to be more complex than mere microbial inflammation. Numerous signaling pathways, particularly inflammatory signaling from a variety of sources, are activated in cholesteatoma and appear to be a key factor in the initiation of cholesteatoma formation [25]. These challenges underscore the need for therapies that target inflammation and its signaling pathways rather than solely addressing infection [14].

In our study, variants were more frequently observed in *IL6*, *TREM1*, *IL1R1*, *IL1A*, *HIF1A*, *ID1*, *RAGE*, and *TNFA* genes in cholesteatoma patients compared with the general UK Biobank population. These genes represent critical downstream mediators or amplifiers of PRR signaling rather than PRRs themselves, suggesting that the pathogenic mechanism may lie in the amplification and persistence of PRR-driven responses.

Variants in *IL6*, *IL1R1*, *IL1A*, and *TNFA* may enhance cytokine production or receptor signaling, thereby reinforcing chronic inflammation and osteoclast-mediated bone resorption. *Trem1* further amplifies TLR-induced cytokine release, intensifying cytokine production and sustaining inflammation [26], while *RAGE*, a receptor for damage-associated molecular patterns, amplifies inflammation via NF-kB pathway activation [27], potentially perpetuating chronic inflammatory cycles. It actually sustains sterile inflammation through the recognition of DAMPs, such as HMGB1 and S100 proteins [28].

The presence of variants in *HIF1A* and *ID1* highlights the role of hypoxia, tissue remodeling, and keratinocyte proliferation in maintaining the inflammatory microenvironment of cholesteatoma [29,30,31]. In more detail, *HIF1A* mediates hypoxia-induced gene expression changes that support angiogenesis and survival in inflamed, hypoxic middle ear tissue [32], while *ID1* regulates the proliferation and differentiation of keratinocytes, potentially contributing to abnormal epithelial growth characteristic of cholesteatoma [33].

Recent genetic and transcriptomic studies support these associations. For example, exome sequencing has identified rare deleterious variants in genes related to inflammatory and tissue remodeling pathways in cholesteatoma patients [34], linking genetic susceptibility to the persistent immune activation seen in disease tissue. Systematic reviews and genome-wide association studies have highlighted the heritability of cholesteatoma and implicate immune-related genes such as *IL6* and *TNFA* in disease risk and recurrence [35,36,37,38]. Taken together, these findings indicate that cholesteatoma pathogenesis may be facilitated not only by the activation of PRRs but also by a genetic predisposition toward a self-perpetuating inflammatory state through enhanced downstream signaling or, in other words, by a prolonged immune signaling amplification and dysregulation.

Our study has several limitations. First, the genes analyzed were selected because they had previously been implicated in the cholesteatoma inflammatory response in either in vitro and/or in vivo studies. It remains possible that other genes related to PRR signaling—such as those involved in downstream pathways of NOD-like or RIG-I-like receptors, which are less well studied in cholesteatoma—may also play a role. Second, not all variants are represented in the UK Biobank dataset. Although the Biobank includes large-scale genotyping with several thousand SNPs, many variants are not covered, and their allele frequencies in both cholesteatoma patients and the general population, therefore, remain unknown.

Another limitation is that, beyond the basic calculation of β-coefficients, numerous potential confounding factors were not accounted for. Recent epidemiological studies using UK Biobank data have reported associations between cholesteatoma and factors such as sex, age, socioeconomic deprivation, and tobacco-related mental and behavioral disorders [39]. In the present study, exploratory regression analyses were therefore performed for selected genetic variants with the highest β-coefficients within genes exhibiting the largest differences in genetic risk scores—variants that contribute most strongly to the overall genetic burden and thus have the greatest potential to introduce confounding. These analyses did not reveal statistically significant associations between the examined polymorphisms and the evaluated demographic, lifestyle, or socioeconomic factors. Given the limited sample size and retrospective design, these findings should be interpreted with caution and warrant validation in larger, independent cohorts. Nonetheless, our method allows for the consistent comparison of genetic burden across genes and highlights those in which polymorphisms are relatively enriched in cholesteatoma patients, potentially contributing to a lower inflammatory threshold and a self-perpetuating inflammatory phenotype. The importance of our findings lies in introducing a different perspective on the role of inflammation in cholesteatoma pathogenesis. Rather than emphasizing pathogen-associated molecular patterns (PAMPs) as the primary drivers, our data suggest that a variety of stressors capable of inducing inflammatory damage may initiate a self-perpetuating inflammatory process. In other words, our results indicate that we need to move the cholesteatoma research field from a simple microbial-centric view to one focused on gene-regulated persistent inflammatory activity.

Previous studies have highlighted the potential contribution of microbial inflammation, including evidence of *TLR4* pathway upregulation [17] and the detection of microbial DNA in virtually all cholesteatoma tissues using advanced methods [40]. However, such approaches cannot distinguish whether microbial involvement represents a primary cause or a secondary phenomenon. By examining genetic variants in inflammatory genes and comparing their frequencies between cholesteatoma patients and the general population, our study supports the possibility that chronic, non-resolving inflammation in cholesteatoma results from the cumulative effect of multiple genetic variants. These variants may lower the threshold for inflammation, allowing not only microbial triggers but also non-microbial stressors—such as smoking, environmental exposures, or intrinsic tissue damage—to drive amplified and self-sustaining inflammation.

This concept is important both for prevention and treatment. From a preventive perspective, future research should focus on identifying and mitigating a wide range of epidemiological stressors that can damage the middle ear, including infections as well as environmental and lifestyle-related factors. In terms of therapy, recent advances in biologics targeting specific inflammatory pathways offer a promising opportunity [10,41]. Repurposing such agents—delivered either topically or systemically—to interrupt the amplification of inflammation could provide a novel therapeutic approach for cholesteatoma, a condition for which no effective medical treatment currently exists. Similar repurposing has already been applied in rhinology and head and neck surgery, suggesting this may be the most promising strategy to develop a medical treatment for cholesteatoma.

## Figures and Tables

**Table 1:** Genetic risk scores for cholesteatoma patients (GRSchol) as well as the total population (GRSpop) of the UK Biobank were calculated for each gene. Then, the differences between them (Δ_GRSchol-GRSpop_) were compared. The genes with the greater differences in genetic risk scores are shown in italic.

Gene	n_SNPs	ΔGRSchol-GRSpop	GRSchol	GRSpop	95% CI (2.5–97.5)
*IL6*	9	0.058526	0.152697	0.094171	0.03260–0.08736
*TREM*	15	0.045138	0.055991	0.010853	0.02846–0.06263
*IL1R1*	14	0.033372	0.044607	0.011234	0.01593–0.05284
*Ila*	10	0.020755	−0.06581	−0.08656	0.00641–0.03601
*HIF1A*	10	0.019255	0.997311	0.978056	0.00772–0.03423
*ID1*	8	0.017261	0.02732	0.010059	0.00554–0.03143
*RAGE*	7	0.014277	0.309153	0.294876	0.00227–0.02815
*TNFa*	5	0.014021	0.043658	0.029637	0.00064–0.03226
*NFKB1*	13	0.007291	−0.29596	−0.30325	0.00252–0.01301
*MINCLE*	10	0.007115	−0.00076	−0.00788	0.00043–0.01612
*TLR4*	8	0.006631	0.016327	0.009696	0.00235–0.01125
*IKK*	11	0.005778	0.061935	0.056157	0.00183–0.01222
*MYD88*	3	0.005543	0.023729	0.018186	0.00043–0.01562
*HMBG*	15	0.005415	0.177825	0.17241	
*MIF*	4	0.005014	−0.0038	−0.00882	
*IL1B*	3	0.004787	0.009527	0.004668	−0.00205–0.01119
*AP1*	1	−0.000562	−0.00785	−0.00841	

**Table 2:** Covariate-adjusted linear regression analyses of *IL6* and *TREM10* polymorphisms.

rs139240442 (*IL6*)			
**Variable**	**β-coefficient**	**95% CI**	***p*** **-Value**
Age (per year)	–0.0007	–0.001 to 0.00006	0.070
Male sex	–0.0060	–0.018 to 0.006	0.344
Ever smoked	0.0049	–0.008 to 0.018	0.470
Townsend deprivation index	–0.0011	–0.003 to 0.001	0.222
Constant	0.0509	0.004 to 0.098	0.032
rs140764737 (*TREM10*)			
**Variable**	**β-coefficient**	**95% CI**	***p*** **-value**
Age (per year)	0.0018	–0.0015 to 0.0052	0.282
Male sex	0.0289	–0.0248 to 0.0826	0.290
Ever smoked	–0.0429	–0.0985 to 0.0127	0.130
Townsend deprivation index	0.0018	–0.0066 to 0.0103	0.674
Constant	–0.0664	–0.2591 to 0.1262	0.497

## Data Availability

The data presented in this study are available through the UK Biobank at https://www.ukbiobank.ac.uk/ (accessed on 5 January 2026) These data were derived from the following resources available in the public domain: https://www.dnanexus.com/.

## Supplementary Materials

The following supporting information can be downloaded at: https://www.mdpi.com/article/10.3390/jpm16020094/s1, Table S1: Based on the UK biobank the β-coefficient of a total of 147 polymorphisms in 17 key inflammatory genes has been calculated.

## Abbreviations

The following abbreviations are used in this manuscript:

PRR	Pattern-recognition receptor
DAMPs	Damage-associated molecular patterns
PAMPs	Pathogen-associated molecular patterns
GRS	Genetic risk score
MYD88	Myeloid differentiation primary response 88
RAGE	Receptor for advanced glycation end products (*gene: AGER*)
NFKB1	Nuclear factor kappa B subunit 1
IL1R1	Interleukin-1 receptor type 1
IL1B	Interleukin-1 beta
IKK Ab	IκB kinase alpha/β
ID1	Inhibitor of DNA binding 1
AP1	Activator protein-1 (JUN/FOS transcription factor complex)
HIF1A	Hypoxia-inducible factor 1-alpha
IL6	Interleukin-6
TLR4	Toll-like receptor 4
HMGB1	High mobility group box 1
TNFA	Tumor necrosis factor-alpha
MIF	Macrophage migration inhibitory factor
MINCLE	Macrophage-inducible C-Type lectin (*CLEC4E*)
TREM	Triggering receptor expressed on myeloid cells
IL1A	Interleukin-1 alpha
