# Supplementary material for: A UK Biobank Study on Genetic Variants in Pattern-Recognition Receptor (PRR) Signaling Indicates Self-Perpetuatin Inflammation of Cholesteatoma
Source: J Pers Med. 2026 Feb 5;16(2):94. doi: 10.3390/jpm16020094 (PMC12941801; doi:10.3390/jpm16020094)
Supplement: Supplementary file 1 [file jpm-16-00094-s001.zip › jpm-3888715-supplementary.pdf]

Supplementary Table S1. Based on the UK biobank the β-coefficient of a total of 147 polymorphisms in 17 key inflammatory genes has been calculated.

gene	Chr	RSID	Reference	Alternate	Variant ID	β coefficient
MYD88	3	["rs545602132"]	C	CGGCGG	3_38138612_C_CGGCGG	0.090153
MYD88	3	["rs4988457"]	C	G	3_38140645_C_G	-0.01748
MYD88	3	["rs4988458"]	T	C	3_38140907_T_C	0.180537
RAGE (AGER)	6	["rs184003"]	C	A	6_32182519_C_A	-0.07106
RAGE (AGER)	6	["rs1035798"]	G	A	6_32183445_G_A	0.040915
RAGE (AGER)	6	["rs35795092"]	G	C	6_32183643_G_C	-0.25638
RAGE (AGER)	6	["rs2070600"]	C	T	6_32183666_C_T	0.015451
RAGE (AGER)	6	["rs115111668"]	C	T	6_32184105_C_T	0.042007
RAGE (AGER)	6	["rs3131300"]	A	G	6_32184157_A_G	0.047352
RAGE (AGER)	6	["rs1800684"]	A	T	6_32184217_A_T	0.169276
NFKB1	4	["rs4648002"]	C	T	4_102537792_C_T	0.247485
NFKB1	4	[]	GCAAAA	GCAAA	4_102584627_CA_C	-0.03786
NFKB1	4	["rs4648039"]	C	T	4_102584804_C_T	-0.05792
NFKB1	4	["rs1609993"]	T	C	4_102593501_T_C	-0.13475
NFKB1	4	["rs4648049"]	C	T	4_102593580_C_T	-0.00977
NFKB1	4	["rs4648050"]	T	C	4_102593584_T_C	-0.01349
NFKB1	4	["rs4648073"]	G	T	4_102597686_G_T	-0.01167
NFKB1	4	["rs4648092"]	TGTAA	T	4_102606442_TGTAA_T	0.195981
NFKB1	4	["rs4648095"]	T	C	4_102606719_T_C	0.004675
NFKB1	4	["rs4648110"]	T	A	4_102612664_T_A	-0.04588
NFKB1	4	["rs4648117"]	C	T	4_102613400_C_T	0.023168
NFKB1	4	["rs3817685"]	C	G	4_102613403_C_G	-0.01337
NFKB1	4	[]	TA	TAA	4_102613583_T_TA	0.002964
IL1R1	2	["rs3917285"]	T	A	2_102164706_T_A	0.010798
IL1R1	2	["rs2228139"]	C	G	2_102165189_C_G	-0.21869
IL1R1	2	["rs3213735"]	GTT	TTT	2_102168698_G_T	-0.09471
IL1R1	2	["rs55639750"]	GTT	GT	2_102168698_GT_G	-0.30271
IL1R1	2	["rs55639750"]	GTT	GTTT	2_102168698_G_GT	0.144031
IL1R1	2	["rs2041747"]	G	A	2_102171949_G_A	0.049283
IL1R1	2	["rs3771200"]	G	A	2_102172314_G_A	0.056425
IL1R1	2	["rs3917306"]	A	G	2_102172379_A_G	-0.27576
IL1R1	2	["rs72820135"]	T	A	2_102172897_T_A	0.480034

IL1R1	2	["rs28362304"]	C	T	2_102174626_C_T	0.296787
IL1R1	2	["rs3917318"]	A	G	2_102176300_A_G	-0.06996
IL1R1	2	["rs112972404"]	T	G	2_102176325_T_G	-0.03675
IL1R1	2	["rs3917320"]	A	C	2_102176415_A_C	0.117506
IL1R1	2	["rs3917322"]	A	G	2_102176786_A_G	-0.03063
IL1B	2	["rs1143639"]	C	T	2_112831216_C_T	0.052994
IL1B	2	["rs1143634"]	G	A	2_112832813_G_A	0.059657
IL1B	2	["rs1143633"]	C	T	2_112832890_C_T	-0.0687
IKK Ab	10	["rs1327575"]	T	C	10_100192759_T_C	0.070171
IKK Ab	10	["rs76731979"]	C	G	10_100193518_C_G	0.012953
IKK Ab	10	["rs2274174"]	C	T	10_100193524_C_T	0.100166
IKK Ab	10	["rs11597086"]	A	C	10_100193948_A_C	0.015831
IKK Ab	10	["rs17878999"]	C	T	10_100194590_C_T	0.053241
IKK Ab	10	["rs41302715"]	T	C	10_100200632_T_C	0.219498
IKK Ab	10	["rs17880383"]	G	A	10_100204555_G_A	-0.0638
IKK Ab	10	["rs200274287"]	GAAC	G	10_100204719_GAAC_G	0.246549
IKK Ab	10	["rs2230804"]	C	T	10_100218126_C_T	0.017718
IKK Ab	10	["rs3763710"]	A	C	10_100218929_A_C	0.078764
IKK Ab	10	["rs17886121"]	G	A	10_100219439_G_A	0.025821
ID1	20	["rs6060263"]	T	C	20_31605307_T_C	0.010723
ID1	20	["rs11545368"]	C	G	20_31605423_C_G	-0.17999
ID1	20	["rs1802548"]	A	G	20_31605574_A_G	0.146477
ID1	20	["rs15817"]	A	G	20_31605735_A_G	-0.03045
ID1	20	["rs200436740"]	G	A	20_31605856_G_A	-0.39897
ID1	20	["rs146555264"]	C	T	20_31605923_C_T	0.39096
ID1	20	["rs8116155"]	G	C	20_31606151_G_C	0.094027
ID1	20	["rs148925564"]	G	A	20_31606190_G_A	0.044332
AP1	1	["rs11688"]	C	T	1_58782321_C_T	-0.07328
HIF1A	14	["rs113439501"]	TG	TGG	14_61695935_T_TG	0.069069
HIF1A	14	["rs1015447"]	AAACG	AAATG	14_61697530_C_T	0.229711
HIF1A	14	["rs17099141"]	G	A	14_61727521_G_A	0.179284
HIF1A	14	["rs11549465"]	C	T	14_61740839_C_T	0.003817
HIF1A	14	["rs61755645"]	A	T	14_61740895_A_T	0.120348
HIF1A	14	["rs4902080"]	T	C	14_61741287_T_C	0.182946
HIF1A	14	["rs141273703"]	A	G	14_61744674_A_G	0.251434
HIF1A	14	["rs60361955"]	CGTGTGT GTGTGTG TGTGT	CGTGTGTG TGTGTGT	14_61744860_CGTGT_C	0.07578
IL-6	7	["rs2069857"]	C	A	7_22727215_C_A	0.650255

IL-6	7	["rs140764737"]	C	T	7_22727607_C_T	0.902275
IL-6	7	["rs2069858"]	G	A	7_22727706_G_A	0.083332
IL-6	7	["rs1524107"]	C	T	7_22728600_C_T	0.11136
IL-6	7	["rs2069843"]	G	A	7_22730375_G_A	0.145561
IL-6	7	["rs2069844"]	C	A	7_22730391_C_A	0.042681
IL-6	7	["rs2069860"]	A	T	7_22731419_A_T	-0.18708
IL-6	7	["rs13306435"]	T	A	7_22731420_T_A	-0.41483
IL-6	7	["rs2069849"]	C	T	7_22731537_C_T	0.134241
TLR4	9	["rs5900307"]	CAAA	CAAAA	9_117704651_C_CA	0.019839
TLR4	9	["rs5900307"]	CAAA	CAAAAA	9_117704651_C_CAA	0.230501
TLR4	9	["rs5900307"]	CAAA	CAA	9_117704651_CA_C	0.000223
TLR4	9	["rs11536871"]	A	C	9_117708220_A_C	0.09065
TLR4	9	["rs78848399"]	A	G	9_117708606_A_G	0.420778
TLR4	9	["rs5030711"]	C	A	9_117712563_C_A	0.273822
TLR4	9	["rs137853920"]	G	A	9_117712970_G_A	0.500248
TLR4	9	["rs4986790"]	A	G	9_117713024_A_G	-0.06731
TLR4	9	["rs5030721"]	G	A	9_117714087_G_A	-0.0402
HMBG	13	[]	GAAA	GAAAA	13_30461330_G_GA	0.047267
HMBG	13	["rs202182303"]	GATC	G	13_30461716_GATC_G	0.147813
HMBG	13	["rs3742305"]	TGTC	TGTG	13_30462505_C_G	-0.03989
HMBG	13	["rs2249825"]	G	C	13_30463766_G_C	-0.01219
HMBG	13	["rs5802568"]	TAAAAA	TAAA	13_30464144_TAA_T	0.153127
HMBG	13	["rs5802568"]	TAAAAA	TAAAA	13_30464144_TA_T	0.128143
HMBG	13	["rs1056130"]	AGGAGA	AGAAGA	13_30464514_G_A	-0.02989
HMBG	13	[]	G	GCGC	13_30464581_G_GCGC	-0.0459
HMBG	13	[]	GCGC	GCGCCGCC GC	13_30464709_G_GCGCCGC	-0.04242
HMBG	13	[]	GCGC	GCGCCGC	13_30464709_G_GCGC	0.484566
HMBG	13	[]	TTGTGTG TGTGTGT G	TTGTGTGT GTGTGTGT GTGTGTG	13_30464761_T_TTGTGTGTG	-0.0017
HMBG	13	[]	TTGTGTG TGTGTGT G	TTGTGTGT GTGTG	13_30464761_TTG_T	-0.04298
HMBG	13	[]	TTGTGTG TGTGTGT G	TTGTGTGT GTGTGTGT GTGTG	13_30464761_T_TTGTGTG	0.222244
HMBG	13	[]	TTGTGTG TGTGTGT G	TTGTGTGT GTGTGTGT GTGTGTGT G	13_30464761_T_TTGTGTGTGTG	-0.34141
HMBG	13	["rs868556252"]	C	T	13_30465180_C_T	0.190442
TNFa	6	["rs3093661"]	G	A	6_31575981_G_A	0.022629
TNFa	6	["rs4645843"]	C	T	6_31576785_C_T	0.455572
TNFa	6	["rs3093664"]	A	G	6_31576865_A_G	0.002158
TNFa	6	["rs542219684"]	C	T	6_31577022_C_T	0.936481
TNFa	6	["rs3093665"]	A	C	6_31577614_A_C	0.01151
MIF	22	["rs112568463"]	A	T	22_23894401_A_T	-0.31019
MIF	22	["rs2096525"]	T	C	22_23894632_T_C	0.001104

MIF	22	["rs33958703"]	CTG	TTG	22_23895002_C_T	0.023699
MIF	22	["rs2070766"]	C	G	22_23895034_C_G	0.004537
MINCLE	12	["rs117557388"]	T	C	12_8537029_T_C	-0.40906
MINCLE	12	["rs112683446"]	C	T	12_8537072_C_T	0.329245
MINCLE	12	["rs113024893"]	G	A	12_8537276_G_A	-0.06376
MINCLE	12	["rs144313874"]	C	G	12_8539759_C_G	-0.03811
MINCLE	12	["rs200497205"]	T	C	12_8539971_T_C	0.516174
MINCLE	12	["rs75520648"]	A	G	12_8540667_A_G	-0.17197
MINCLE	12	["rs4620776"]	G	A	12_8540737_G_A	-0.00716
MINCLE	12	["rs11834597"]	T	C	12_8540822_T_C	0.002146
MINCLE	12	[]	TTCTC	TTCTCTCTC	12_8540823_T_TTCTC	-0.00049
MINCLE	12	[]	TTCTC	TTCTCTC	12_8540823_T_TTC	-0.03485
TREM	6	["rs2234243"]	C	T	6_41276253_C_T	0.089522
TREM	6	["rs2234242"]	G	A	6_41276282_G_A	0.104016
TREM	6	["rs200624147"]	GA	G	6_41279514_GA_G	0.262869
TREM	6	["rs111838034"]	AT	AA	6_41279526_T_A	-0.38264
TREM	6	["rs79918075"]	C	G	6_41279542_C_G	-0.5278
TREM	6	["rs6916548"]	A	G	6_41279758_A_G	-0.25042
TREM	6	["rs6940092"]	T	C	6_41280026_T_C	0.002406
TREM	6	["rs115985878"]	A	G	6_41280053_A_G	0.060136
TREM	6	["rs6458211"]	G	T	6_41280147_G_T	-0.50105
TREM	6	["rs77643173"]	G	A	6_41280419_G_A	-0.57912
TREM	6	["rs111673840"]	G	A	6_41280496_G_A	-0.16025
TREM	6	["rs2234239"]	C	T	6_41282441_C_T	0.062022
TREM	6	["rs2234237"]	T	A	6_41282728_T_A	0.062414
TREM	6	["rs112617855"]	A	AT	6_41282771_A_AT	-0.5781
TREM	6	["rs139240442"]	T	C	6_41282789_T_C	0.908974
Ila	2	["rs3783550"]	G	T	2_112775308_G_T	-0.01656
Ila	2	[]	GGTGTGTGT	GGTGTGTGTGTGTGT	2_112778193_G_GGTGTGT	0.768736
Ila	2	[]	GGTGTGTGT	GGTGT	2_112778193_GGTGT_G	-0.10107
Ila	2	[]	GGTGTGTGT	GGTGTGT	2_112778193_GGT_G	-0.03328
Ila	2	[]	GGTGTGTGT	GGTGTGTGTGT	2_112778193_G_GGT	-0.3825
Ila	2	[]	GGTGTGTGT	GGTGTGTGTGTGT	2_112778193_G_GGTGT	-0.24661
Ila	2	["rs17561"]	GTGCTGA	GTGATGA	2_112779646_C_A	0.019428
Ila	2	["rs2856841"]	A	G	2_112779762_A_G	0.014847
Ila	2	["rs3783531"]	C	T	2_112781669_C_T	0.75298
Ila	2	["rs1609682"]	G	T	2_112782628_G_T	-0.02831
